# Prevalence of ocular surface disease symptoms and risk factors in group of university students in Monterrey, Mexico

**DOI:** 10.1186/s12348-016-0114-z

**Published:** 2016-11-18

**Authors:** Manuel Garza-León, Miguel Valencia-Garza, Bernardo Martínez-Leal, Pablo Villarreal-Peña, Hernán Gerardo Marcos-Abdala, Ana Lucía Cortéz-Guajardo, Arturo Jasso-Banda

**Affiliations:** 1 Dirección de Ciencias Médicas de la División de Ciencias de la Salud, Universidad de Monterrey, Monterrey, Nuevo León México; 2 Fundación Destellos de Luz IBP, San Pedro Garza García, Nuevo León México; 3 Department of Statistical Science, University College London, London, WC1E6BT UK; 4 Hidalgo # 2425, consultorio 706, Colonia Obispado, Monterrey, Nuevo León México

**Keywords:** Ocular surface disease, Prevalence, Risk factors, OSDI questionnaire, Dry eye, College students, Mexico, University students

## Abstract

**Background:**

The objective of this study was to determine the prevalence of symptoms of ocular surface disease and its relationship with associated risk factors in students from the University of Monterrey using Ocular Surface Disease (OSDI) questionnaire.

**Methods:**

A cross-sectional survey was conducted between October and December 2014 to assess the prevalence and risk factors for ocular surface disease in a group of students from Universidad de Monterrey in Monterrey, Mexico. The severity of the disease was measured via the Ocular Surface Disease Index (OSDI) questionnaire.

**Results:**

The OSDI average value was 26.85 ± 20.79 points, with 70.4% of students (579) had OSDI score higher than 12 points. Women had ocular surface disease 1.63 times more than men (OR 1.29, 95% CI 1.13,1.48). Students who used ophthalmic drops have an OR 2.00 (95% CI 1.65,2.40), and students who smoke have an OR 1.24 (95% CI 1.06,1.46). Use of contact lenses, hours in front of computer or history of refractive surgery has low-estimated effect on the probability of presenting an ocular disease.

**Conclusions:**

University students have a prevalence of 70.4% of ocular surface disease (OSD). OSD was associated with gender (women have a higher prevalence), smoking and the use of eye drops. A program to modify these risk factors to reduce the prevalence is needed.

## What is new

- We found a high prevalence of ocular surface disease symptoms in university students.
- Female gender, smoking and use of eye drops are the most important risk factors of ocular surface disease symptoms in university students.
- Awareness campaigns of the harmful effects of smoking in the eyes should institute in the university.

## Background

In 2007, the International Dry Eye Workshop (DEWS) dry eye as ‘a multifactorial disease of the tears and ocular surface that results in symptoms of discomfort, visual disturbance and tear film instability, with potential damage to the ocular surface’ [1]. The DEWS reported that dry eye is one of the most prevalent eye diseases and a main reason to seek eye care in patients with advanced age [2]. There are several risk factors that influence the development of dry eye disease, such as external conditions involving low humidity [3, 4], urban pollution and high levels of CO_2_. Other factors are related to personal activities such excessive use of video display units (VDU) [5], smoking [6, 7], use of contact lenses [5], refractive surgery [8] and use of ocular drops [9].

There have been several studies around the world aimed to determine the prevalence of dry eye disease with results ranging from 5 to 30% in people over 50 years [2, 10]. This variability is due to study definition of the disease, variations in the diagnostic methods used and the population studied. To date, there are few studies evaluating the prevalence of dry eye in young people. Uchino et al. studied 3433 school students through a short questionnaire for dry eye syndrome by Schaumberg et al. and found severe dry eye symptoms in 21% of men and 24% of women [11].

At the present time, symptom questionnaires are among the most repeatable of the commonly used diagnostic tests for dry eye [2]. These questionnaires provide a more integrated view of the clinical condition over time. Also, they are easy to apply to large populations at a lower cost. There are over 15 different questionnaires to diagnose dry eye. Most of them only assess the symptoms associated with the disease. The Impact of Dry Eye in Everyday Life, Ocular Surface Disease and Ocular Surface Disease Index questionnaires assess dry eye’s impact on the quality of life as well as the symptoms associated with it [6–8].

The Ocular Surface Disease Index questionnaire (OSDI) is a widely used method in epidemiological studies. It consists of 12 questions on symptomatology and the impact on the quality of life of respondent. Each question has a score on a scale 0 to 4. Overall OSDI score ranges from 0 to 100. Based on this result, it can categorise patients as follows: normal ocular surface (0–12 units), mild ocular surface disease (13–22), moderate (23–32) and severe (33–100) [12]. To carry out our study, we used a validated version of the OSDI questionnaire in Spanish language [13].

The objective of this study was to determine the prevalence of symptoms of ocular surface disease and its relationship with associated risk factors in students from the University of Monterrey using Ocular Surface Disease (OSDI) questionnaire.

## Methods

We conducted a cross-sectional observational survey study at University of Monterrey to assess dry eye symptoms (Ocular Surface Disease Index) and their associated risk factors such as smoking status, history of refractive surgery, use of contact lens and ophthalmic drops. We surveyed students between October and December 2014.

This study was conducted under the approval of the authorities and Ethics Committee of University of Monterrey and adhered to the tenets of the Declaration of Helsinki.

### Sampling

The target population were all undergraduate and graduate students officially registered at University of Monterrey for the academic year 2014–2015. We obtained the sampled population via stratified sampling. We defined the strata according to the different schools (namely School of Health Sciences; School of Education and Humanities; School of Art, Architecture and Design; School Law and Social Sciences; School of Engineering and Technology; and School of Business) in which the university is divided. The questionnaire was applied between classes, inside the classroom.

An informed consent was obtained from all participants after providing a brief explanation of the study. The participants completed the OSDI questionnaire by self-administration.

### Sample size

We calculated a sample size of 813 students based on an assumed prevalence of 24% (Uchino et al. [11]), an error of ±2.75% and design effect of 1.0.

### Statistical analysis

We determined the prevalence of symptoms of ocular surface disease as well as the relationship among the risk factors and the students’ OSDI score. We considered the following risk factors: (1) gender, (2) smoking status, (3) history of refractive surgery, (4) use of contact lenses and (5) use of ophthalmic drops.

As mentioned before, the OSDI score is bounded between 0 and 100, where 0 represents a healthy eye in terms of ocular surface diseases whilst 100 represents a patient with severe symptoms of ocular surface disease and could be categorised in normal ocular surface (0–12 units), mild ocular surface disease (13–22), moderate (23–32) and severe (33–100) [12].

Bounded outcomes usually display skewed distributions. This feature makes inappropriate analysis through methods that assume Gaussianity. For this reason, we use another approach: we transform the values so they lie in the (0,1) interval. Variables that are defined in (0,1) can be modelled using the Beta distribution. In our case, we can change the scale of the OSDI score from 0–100 to 0–1 without losing the meaning of the original values.

To quantify the association of the OSDI score with the risk factors, we model the mean of the Beta distribution as a regression function of these factors. This model is called Beta regression [14]. Given this relationship, we estimate how each risk factor (holding the rest constant) affects the mean OSDI score of our population of interest.

In addition to estimating the effects of the risk factors on the mean OSDI score, we predicted the mean OSDI score of students with different characteristics and make recommendations based on these values.

We used statistical software R (R Core Team, 2015) to analyse our results and Stan (Stan Development Team. 2016. RStan: the R interface to Stan, Version 2.9.0. http://mc-stan.org) to fit our Bayesian Beta regression model.

## Results

We got responses from 823 students (95.7%) out of 860 students selected to be part of the study. From the participants, 492 (59.8%) were female and 331 (40.2%) male, with an average age of 21.38 ± 1.79 years (range 17–33). Women had an average age of 21.04 ± 1.59 years and men 21.89 ± 1.94 years, with a statistically significant difference (*p* < 0.001), demographic data are presented in Table 1. Almost 80% of students responded to all the questions (12), followed by 8.4% who responded 11 and 4% with 8 responses, the lowest number of responses was 5 in 3.5% of the questionnaires.

**Table 1:** Gender and age distribution according to disease severity

Group	Age	Total (% of total)	Female (%) *n* = 492	Male (%) *n* = 331
No disease	21.33 ± 1.87	244 (29.6)	124 (25.2)	120 (36.3)
Mild disease	21.38 ± 1.62	163 (19.9)	95 (19.3)	68 (20.5)
Moderate disease	21.50 ± 1.73	122 (14.8)	75(15.2)	47 (14.2)
Severe disease	21.38 ± 1.83	294 (35.7)	198 (40.2)	96 (29)

**Fig. 1:**
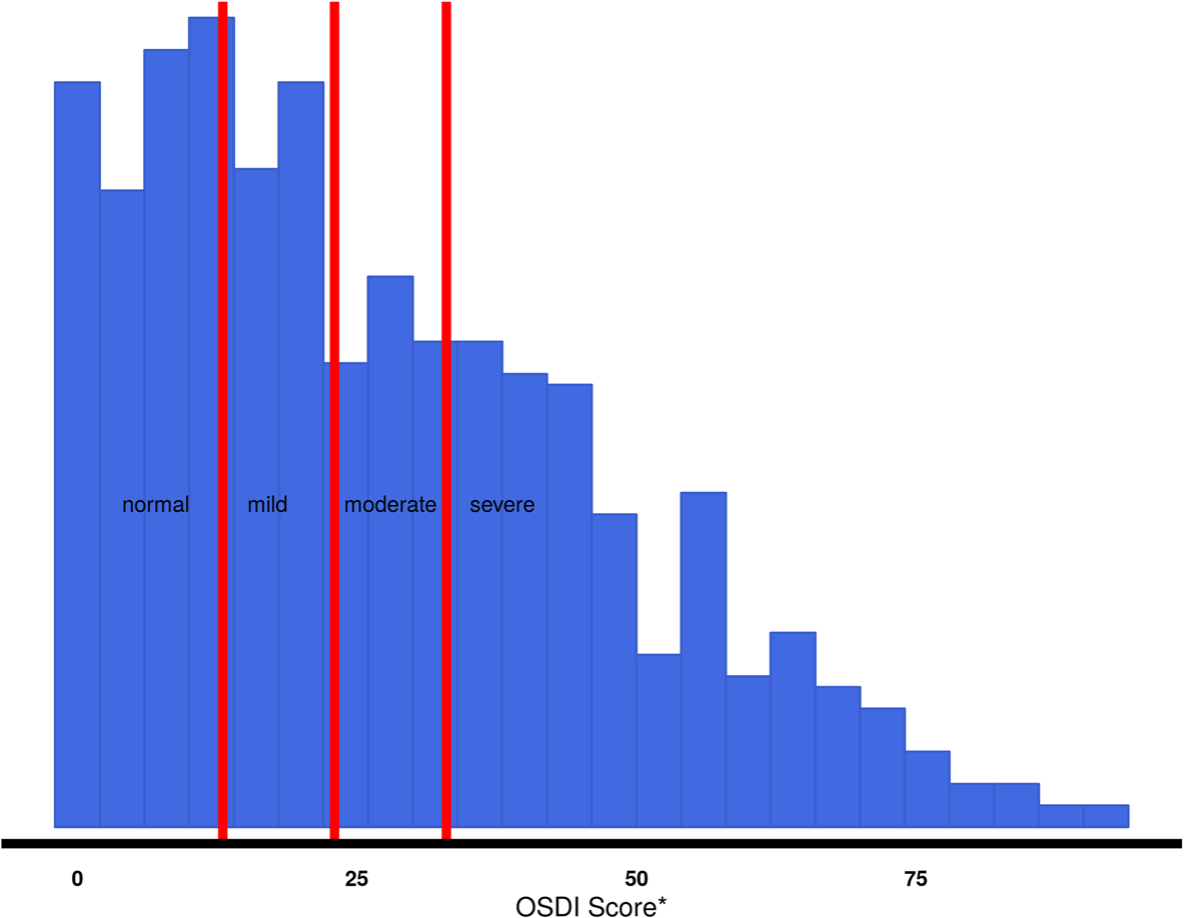
This figure shows the OSDI general score, and with the *red lines*, we separate the severity of the ocular surface disease symptoms found in our students

The average OSDI score was 26.85 ± 20.79 units. Five hundred seventy-nine participants (70.4%) presented ocular surface disease symptoms (OSDI > 12). There were no age differences among participants with respect to severity of OSD symptoms. Figure 1 shows the distribution of *OSDI score* and how these values are divided with respect to the aforementioned categorisation.

Table 2 shows the estimated effects of the risk factors that we obtained via our Beta regression model. Women are likely to present a higher mean OSDI score than men (OR 1.29, 95% CI 1.13,1.48).

**Table 2:** Estimated effects with odds ratio of the risk factors

Risk factor	OR (95% CI)
Gender	
Male	–
Female	1.29 (1.13,1.48)
Smoking habit	
Non-smoker	–
Smoker	1.24 (1.06,1.46)
Hours in front of a computer	0.82 (0.72,0.93)
Refractive surgery	
No	–
Yes	0.82 (0.61,1.09)
Eye drops user	
No	–
Yes	2.00 (1.65,2.4)
Contact lens user	
No	–
Yes	1.12 (0.84,1.48)

One hundred seven participants (13%) reported some types of eye drop use. The types of ophthalmic drop used are lubricants (99 students), anti-allergic treatment (2 students), anti-glaucoma medication (1 student) and 5 students that did not specify the type of drop they use. The odds ratio of eye drop users against those that do not use them is 2, all other variables held fixed.

Twenty-three percent of students reported smoking. There were more male smokers (28.4%) than female (19.7%) (*p* = 0.003). Those students that smoke present a higher mean OSDI score compared to those that do not smoke (OR 1.24, 95% CI 1.06,1.46).

Forty-seven participants had history of refractive surgery. The average time elapsed since the procedure was of 3.67 ± 2.16 years. The mean OSDI score in participants who underwent refractive surgery was 24.53 ± 19.37 and 26.99 ± 20.88 for those without history of surgery. We found that the estimated effect of this factor on the mean OSDI score is low. This suggests that the students’ OSDI score is unaffected by their history of refractive surgery.

We estimated that using contact lens has also a low effect on the mean OSDI score, after adjusting for the presence of the other factors. This low effect might be caused by the small number of participants (5.3% of surveyed students) that use contact lens.

Finally, we see that as the hours in front of a computer screen increase the mean OSDI score decreases (OR 0.82, 95% CI 0.72,0.93).

Based on the evidence gathered, smoking habits, use of eye drops and the hours spent in front of a computer influence UdeM students’ OSDI score. In particular, smoking and using ophthalmic drops increase the chances of having a higher mean OSDI score than those students that do not smoke and do not use eye drops. On the other hand, increasing in 1 h time spent in front of a computer screen increase the chances of having a lower mean OSDI score.

Using our estimations, we wish to predict the expected OSDI score of students with different characteristics. In particular, we want to find the expected OSDI score for the following:

1. A female student that does not smoke and does not use eye drops (*fa*).
2. A female student that smokes and does not use eye drops (*fb*).
3. A female student that smokes and uses eye drops (*fc*).
4. A male student that does not smoke and does not use eye drops (*ma*).
5. A male student that smokes and does not use eye drops (*mb*).
6. A male student that smokes and uses eye drops (*mc*).

For all these, we also assume that the students described (1–6) have not undergone refractive surgery and that the hours spent in front of a computer screen equal to the mean of the total number of hours recorded.

**Fig. 2:**
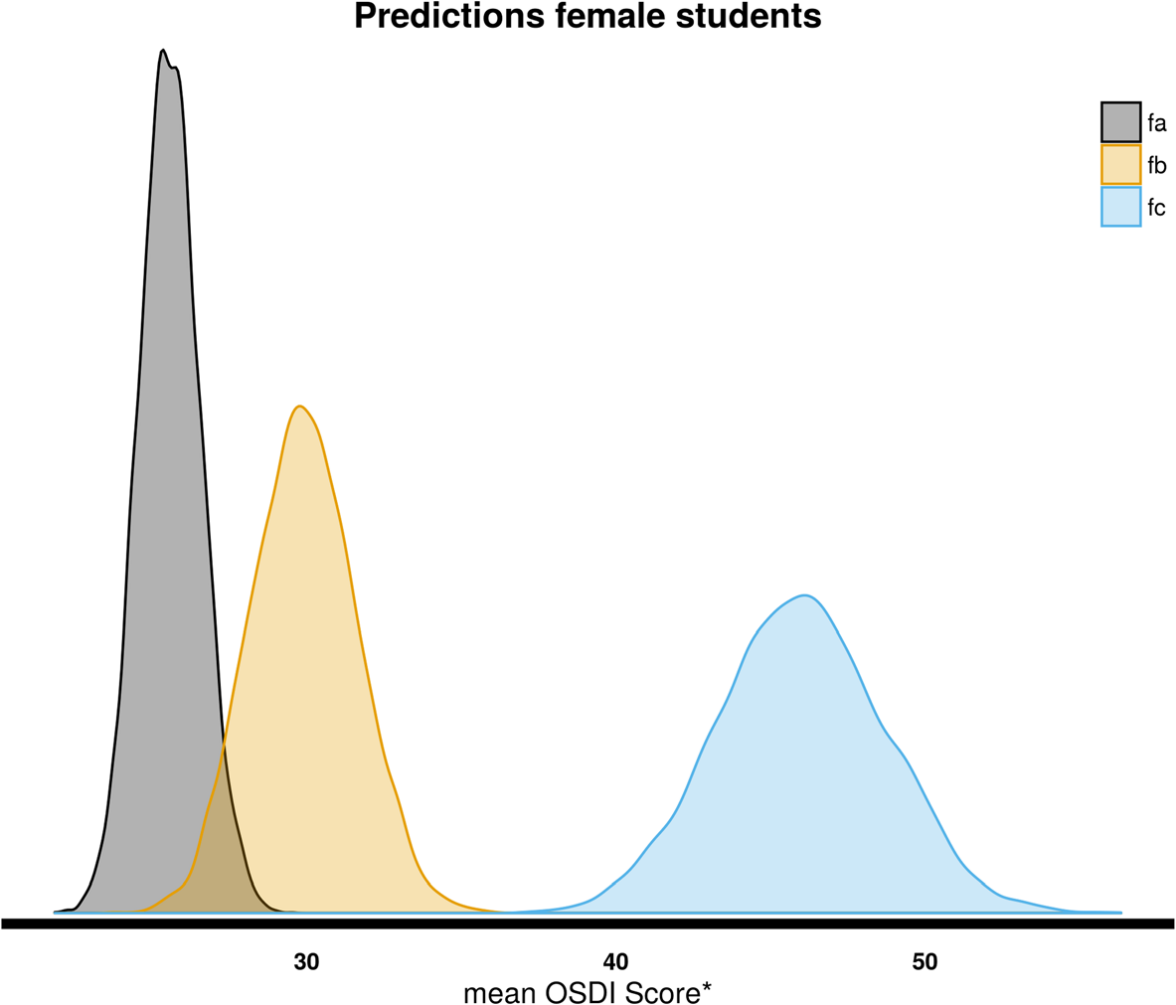
The frequency of female student that does not smoke and does not use eye drops (*fa*) was high, and the mean OSDI score was the lowest (26 points). In the opposite side, we can see the female student that smokes and uses eye drops (*fc*) with the higher OSDI score (46 points), and the lowest frequency; and between both female students that smoke and do not use eye drops (*fb*) with an OSDI score of 30 points

**Fig. 3:**
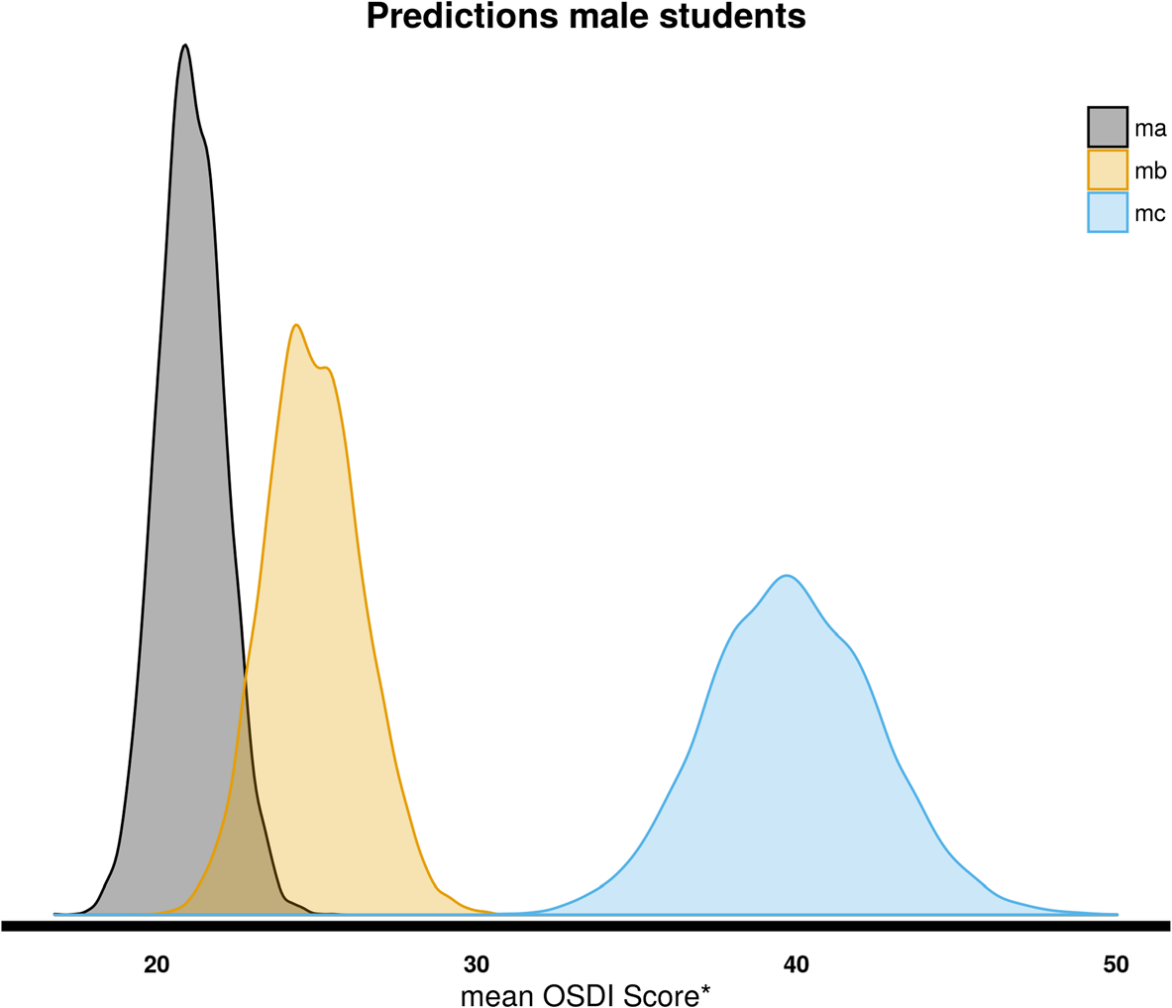
Same with the female students although with lower OSDI scores, male students that do not smoke and do not use eye drops (*ma*) has the highest frequency and the lowest mean OSDI score (21 points); on the other side are the male student that smokes and uses eye drops (*mc*) with the higher OSDI score (40 points) and the lowest frequency: and between both male students that smoke and do not use eye drops (*mb*) with an OSDI score of 25 points

Figures 2 and 3 show the estimated distributions of the predicted mean OSDI scores, and Table 3 shows the mean values of these predictions as well as their 95% credible intervals.

**Table 3:** Estimated distributions of the predicted mean OSDI score

Student	Mean OSDI score (95% CI)—disease category
A female student that does not smoke and does not use eye drops (fa)	26 (24,28)—mild
A female student that smokes and does not use eye drops (fb)	30 (27,33)—moderate
A female student that smokes and uses eye drops (fc)	46 (41,51)—severe
A male student that does not smoke and does not use eye drops (ma)	21 (19,23)—normal/mild
A male student that smokes and does not use eye drops (mb)	25(22,28)—mild/moderate
A male student that smokes and uses eye drops (mc)	40 (35,45)—severe

We see that on average, women have higher OSDI scores. Smoking increase the expected OSDI score of both women and men students. The mean OSDI score increases further if the students uses ophthalmic drops.

## Discussion

The unconditional mean OSDI score was 26.85 ± 20.79 points. This value is lower than that reported by Özcura and colleagues [15] (34.77 ± 22.37) who studied 68 patients 18 years or older that did not have a previous diagnosis of dry eye, eye surgery, pterygium or obstruction of the nasolacrimal pathway. Unlü et al. [16] in 35 computer users (average age of 29.09 ± 6.73 years) reported an OSDI score of 37.12 ± 19.05.

We estimated the following prevalence of ocular surface disease symptoms: 70.4%, 19.9% mild, 14.8% moderate and 35.7% severe disease. For severe dry eye disease, we found value higher than the one estimated by Uchino et al [17]. They found severe symptoms in high school students in Japan (21% of men and 24.4% women), although these authors used the questionnaire developed by Schaumberg et al [18]. This same questionnaire was used in Zhang et al. [19]. They focused on students from senior high school in China. They found a prevalence of 23.7% of severe symptoms. From this stratum, 58.6% had symptoms of constant or often irritation which is similar to what we found with OSDI questionnaire.

Like other studies of dry eye and ocular surface disease [11, 17, 20], we found higher prevalence and more severe symptoms in women than in men with an OR of 1.29 (95% CI 1.13,1.48). This risk factor persists even in patients who smoke and use eye drops.

Our OSDI values were higher in students who smoke (30.52 ± 21.26 points) compared with non-smokers (25.74 ± 20.53 points), this is consistent with epidemiological studies that evaluate the effects of smoking in ocular health. It has been shown that smoking is linked to an increased risk of developing cataracts, age-related macular degeneration [21], glaucoma [22] and among others. The Beaver Dam study related active smoking (or smoking history) with a higher prevalence of dry eye [23]. Other alterations in the ocular surface related to smoking habits are decreased tear breakup time [6], conjunctival and corneal hypoesthesia [7] and changes in the composition of the tear [24].

Although dry eye is the most common complication of refractive surgery [8, 25–27], this usually lasts less than 6 months [25] and the peak of symptoms occurs between the first week and 3 months [8]. Students without refractive surgery have higher OSDI score than those who have had the surgery, this is possibly related to the average time of surgery was significantly higher (3.67 ± 2.16 years) than the 6 months that generally takes the corneal re-innervation, and the year in which the pre-operative corneal sensitivity is recovered [28, 29].

In our study, we found that contact lens users (5.3% of the students) had OSDI results higher than non-users (33.63 ± 24.46 and 26.46 ± 20.52 points, respectively), although we found that this factor had a low effect on the probability of developing dry eye disease. This is different from those reported by Uchino et al [11]. This could be due to the small number of students using contact lens (CL) (44) in our sample, and the high prevalence of symptoms of ocular surface disease found.

The use of ophthalmic drugs was significantly associated with an increased rate of ocular surface symptoms. In our study, 13% of the students use any eye drops and these patients had much higher OSDI values than non-users. This coincides with the findings reported in Zhang et al [19] with students’ in ‘senior high school’ in China.

Although other studies [16, 17, 30–32] linked the use of video displayers with the development of dry eye, our study failed to confirm this finding. Same as reported by Unlu et al. [16], they did not find a statistically significant correlation between time spent using computers each day and the mean OSDI, TBUT and Schirmer’s test scores in a group of young patients; our results report that students who spend more time in front of the computer had fewer symptoms of ocular surface disease; this could be due to several factors: first is that the position of the computer screen was not evaluated and lowering the screen level has been reported to reduce the overall ocular surface area and to decrease the tear evaporation [33], or perhaps the time they spend on the computer are in areas with good moisture or finally that the time is not continuous and that allows better dynamics in eye blinking and less evaporation of the tear film.

Finally, other factors that may influence the high prevalence of symptoms of ocular surface disease in our students are the university’s educational level that was associated with a higher prevalence of dry eye with an OR of 1.6, according to a report by Ahn et al [34] on the results of the national survey of health and nutrition in South Korea, and the use of computer equipment [16, 17, 35] are in continuous use in college students.

The present study has some limitations. The most important limitation is related to the only use of questionnaire to diagnose the ocular surface disease. Many studies reported that the correlations between symptoms measured by questionnaires and clinical findings are poor [36, 37]. We did not study in depth the use of contact lens. Thus, we were unable to discover how use and care patterns (e.g. frequency of replacement, type of cleaning solutions) affect dry eye symptoms. Other factors such as environmental conditions [3, 4, 38–41], stress levels [34] and autoimmune diseases [34, 42, 43] were not included.

## Conclusions

In conclusion, we found a prevalence of 70.4% of ocular surface disease in the University of Monterrey’s students’ population. We found that ocular surface disease was associated with gender (women have higher prevalence), smoking and the use of ophthalmic eye drops, so we suggest that awareness campaigns of the harmful effects of smoking should made in the university.
